# Genome‐wide significant schizophrenia risk variation on chromosome 10q24 is associated with altered *cis*‐regulation of *BORCS7*, *AS3MT*, and *NT5C2* in the human brain

**DOI:** 10.1002/ajmg.b.32445

**Published:** 2016-03-22

**Authors:** Rodrigo R. R. Duarte, Claire Troakes, Matthew Nolan, Deepak P. Srivastava, Robin M. Murray, Nicholas J. Bray

**Affiliations:** ^1^ Department of Basic and Clinical NeuroscienceInstitute of Psychiatry, Psychology and NeuroscienceKing's College LondonLondonUnited Kingdom; ^2^ Department of Psychosis StudiesInstitute of Psychiatry, Psychology and NeuroscienceKing's College LondonLondonUnited Kingdom; ^3^ MRC Centre for Neuropsychiatric Genetics and GenomicsCardiff University School of MedicineCardiffUnited Kingdom

**Keywords:** GWAS, gene expression, functional genetics, allele‐specific expression

## Abstract

Chromosome 10q24.32‐q24.33 is one of the most robustly supported risk loci to emerge from genome‐wide association studies (GWAS) of schizophrenia. However, extensive linkage disequilibrium makes it difficult to distinguish the actual susceptibility gene(s) at the locus, limiting its value for improving biological understanding of the condition. In the absence of coding changes that can account for the association, risk is likely conferred by altered regulation of one or more genes in the region. We, therefore, used highly sensitive measures of allele‐specific expression to assess *cis‐*regulatory effects associated with the two best‐supported schizophrenia risk variants (SNP rs11191419 and indel ch10_104957618_I/rs202213518) on the primary positional candidates *BORCS7*, *AS3MT*, *CNNM2*, and *NT5C2* in the human brain. Heterozygosity at rs11191419 was associated with increased allelic expression of *BORCS7* and *AS3MT* in the fetal and adult brain, and with reduced allelic expression of *NT5C2* in the adult brain. Heterozygosity at ch10_104957618_I was associated with reduced allelic expression of *NT5C2* in both the fetal and adult brain. Comparisons between cDNA ratios in heterozygotes and homozygotes for the risk alleles indicated that *cis*‐effects on *NT5C2* expression in the adult dorsolateral prefrontal cortex could be largely accounted for by genotype at these two risk variants. While not excluding effects on other genes in the region, this study implicates altered neural expression of *BORCS7*, *AS3MT*, and *NT5C2* in susceptibility to schizophrenia arising from genetic variation at the chromosome 10q24 locus. © 2016 The Authors. *American Journal of Medical Genetics Part B: Neuropsychiatric Genetics* Published by Wiley Periodicals, Inc.

## INTRODUCTION

**Figure 1:**
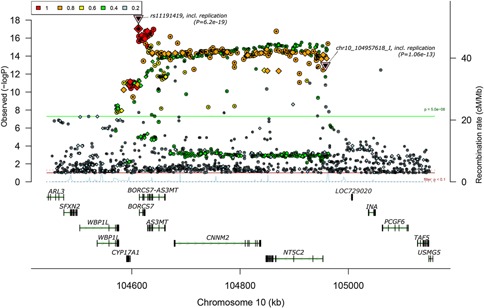
Genetic association with schizophrenia in a region of strong linkage disequilibrium on chromosome 10q24.32‐q24.33. Plot generated by Ricopili (http://www.broadinstitute.org/mpg/ricopili/) using the PGC_SCZ52_may13 dataset described in the Schizophrenia Working Group of the Psychiatric Genomics Consortium [2014] study. Positions of rs11191419 and ch10_104957618_I are indicated by triangles. The threshold of genome‐wide significance (*P* < 5 × 10^−8^) is indicated by a green horizontal line. Color key indicates r^2^ between the variant at the locus showing most significant association with schizophrenia (rs11191419) and other variants in the region. [Color figure can be seen in the online version of this article, available at http://wileyonlinelibrary.com/journal/ajmgb]

Chromosome 10q24.32‐q24.33 is one of the best‐supported genetic risk loci to arise from large‐scale genome‐wide association studies (GWAS) of schizophrenia [Schizophrenia Psychiatric Genome‐Wide Association Study Consortium, 2011; Ripke et al., 2013; Schizophrenia Working Group of the Psychiatric Genomics Consortium, 2014]. Variation at this locus also exhibits genome‐wide significant association with the five disorders included in the Psychiatric Genomics Consortium combined [Cross‐Disorder Group of the Psychiatric Genomics Consortium, 2013], suggesting that it increases susceptibility to psychiatric disorders in general. However, like many other loci identified by schizophrenia GWAS, extensive linkage disequilibrium in the region results in association signals spanning multiple genes (Fig. 1), making it difficult to predict the actual susceptibility gene(s) at the locus.

As with the majority of genome‐wide significant signals for schizophrenia, the chromosome 10q24 variants exhibiting strongest evidence for association are in non‐coding sequence [Schizophrenia Working Group of the Psychiatric Genomics Consortium, 2014]. These variants do not appear to index variation influencing protein structure, and are therefore likely to confer risk for schizophrenia through effects on the expression of one or more genes in the region. Measures of allele‐specific expression provide a powerful means of assessing such *cis*‐regulatory influences, because they allow the level of gene expression from chromosomes carrying the risk and non‐risk alleles of a given variant to be compared simultaneously within individual samples [Bray et al., 2003a]. This approach typically makes use of exonic (i.e., expressed) single nucleotide polymorphisms (SNPs) in genes of interest as allele‐specific tags, allowing the RNA transcribed from each parental chromosome to be distinguished and relatively quantified in individual heterozygotes [Yan et al., 2002]. A major advantage of this method over traditional expression quantitative trait loci (eQTL) approaches based on total gene expression is that it effectively controls for tissue variables such as RNA quality as well as confounding effects of other genetic and environmental variables, since these influences will usually act on both alleles to the same extent [Bray et al., 2003b].

In order to identify genes that are differentially *cis*‐regulated in association with schizophrenia risk variants on chromosome 10q24 (and therefore genes at the locus that potentially confer susceptibility to the disorder), we assessed genotypic effects on the allele‐specific expression of the genes encompassed by the strongest schizophrenia association signal: *BORCS7* (formerly *C10ORF32*), *AS3MT*, *CNNM2*, and *NT5C2* (Fig. 1). As *cis*‐effects on gene expression can be specific to developmental stage [Hill and Bray, 2012; Tao et al., 2014] and brain region [Buonocore et al., 2010; Gibbs et al., 2010; Ramasamy et al., 2014], we examined effects in the human fetal brain as well as in three adult brain regions implicated in the pathophysiology of schizophrenia: dorsolateral prefrontal cortex (DLPFC), hippocampus, and caudate.

## MATERIALS AND METHODS

### Brain Samples

Ethical approval for this study was provided by The Joint South London and Maudsley and The Institute of Psychiatry NHS Research Ethics Committee (REF: PNM/12/13‐102). Post‐mortem human brain tissue from 116 unrelated adults (mean age at death: 72 years; range: 18–102 years) was obtained from the London Neurodegenerative Diseases Brain Bank (UK). All subjects were free from psychiatric or neurological diagnosis at the time of death. Whole brain from 95 second trimester human fetuses (13–23 post‐conception weeks) was provided by the MRC—Wellcome Trust Human Developmental Biology Resource (UK). The demographics of samples assayed for each candidate gene are provided in Supplementary Table S1. Genomic DNA was initially extracted from all samples using standard phenol/chloroform procedures, and was used to genotype for the schizophrenia risk variants and the exonic SNPs used to assay the allele‐specific expression of each gene. Total RNA was extracted from each brain sample using Tri‐Reagent (Life Technologies, Paisley, UK), according to manufacturer's instructions. RNA samples were treated with TURBO DNase (Life Technologies) prior to reverse transcription and did not yield a PCR product in the absence of a reverse transcription step. Approximately 1 μg of total RNA was reverse transcribed with SuperScript III and random decamers (Life Technologies). Resulting cDNA was diluted 1:7 prior to use.

### Genotyping

In order to identify heterozygotes informative for the allele‐specific expression assays, all samples were initially genotyped for the exonic (expressed) SNPs rs4917985, rs1046778, rs2275271, and rs3740387, tagging *BORCS7*, *AS3MT, CNNM2*, and *NT5C2* transcripts, respectively. Genotyping of exonic SNPs was performed by single base primer extension using SNaPshot® chemistry (Life Technologies). All 116 adult and 95 fetal samples were also genotyped for schizophrenia risk SNP rs11191419 and indel ch10_104957618_I (rs202213518). These were selected for study because they are the two independent (r^2^ < 0.2) variants at the chromosome 10q24 locus showing most significant association with schizophrenia (rs11191419, *P* = 6.2 × 10^−19^; ch10_104957618_I, *P* = 1.06 × 10^−13^) in the largest GWAS of the disorder published to date [Schizophrenia Working Group of the Psychiatric Genomics Consortium, 2014] (Fig. 1). SNP rs11191419 was genotyped by single base primer extension using SNaPshot® chemistry (Life Technologies). The ch10_104957618_I indel was genotyped by Sanger sequencing using BigDye Terminator v3.1 (Life Technologies) in the forward and reverse direction. Primer sequences are provided in Supplementary Table S2. We had previously genotyped all adult samples for rs7085104 and rs11191580, two chromosome 10q24 SNPs reported as genome‐wide significant in earlier GWAS of schizophrenia [Schizophrenia Psychiatric Genome‐Wide Association Study Consortium, 2011; Ripke et al., 2013], using SNaPshot® primer extension (Life Technologies). SNP rs7085104 is in strong linkage disequilibrium (LD) with rs11191419 in our samples (r^2 ^= 0.79), while SNP rs11191580 is in strong LD with ch10_104957618_I (r^2 ^= 0.82), suggesting that they index the same functional risk variation. There was no significant (*P* < 0.05) deviation from Hardy‐Weinberg equilibrium in the genotype distribution of any of the genotyped variants.

### Assessment of Allele‐Specific Expression

To investigate variable *cis*‐effects on the expression of genes at the chromosome 10q24 locus, we used a common exonic SNP in each gene to distinguish the RNA transcribed from each chromosomal copy. Expressed SNPs rs4917985, rs1046778, rs2275271, and rs3740387 were used to tag RNA transcripts for *BORCS7*, *AS3MT*, *CNNM2*, and *NT5C2*, respectively. The allele‐specific expression assay was performed using brain cDNA alongside the corresponding genomic DNA from the same subjects. Sequences containing the exonic tag SNPs were PCR‐amplified using primers based on single exon sequence, each producing the same amplicon from both cDNA and genomic DNA (Supplementary Table S2). Four technical replicates for each cDNA and genomic DNA sample were assayed for each expressed SNP, with one H_2_O‐negative control on each plate. PCR products were treated with shrimp alkaline phosphatase and exonuclease I (New England Biolabs, Hitchin, UK) to inactivate nucleotides and primers for downstream steps. Alleles of each expressed SNP were discriminated and relatively quantified by SNaPshot® primer extension (Life Technologies) using extension primers detailed in Supplementary Table S2. Reaction products were electrophoresed on an Applied Biosystems 3130xl Genetic Analyzer and peak heights of allele‐specific extended primers were determined using GeneMarker software (SoftGenetics, State College, PA). Peak heights representing the relative abundance of each allele were used to calculate an allele ratio for each reaction. Allele ratios were calculated for each expressed SNP by dividing peak height for the expressed allele that is usually in phase with the risk alleles of rs11191419/ch10_104957618_I by the peak height of the expressed allele that is usually in phase with the non‐risk alleles of these variants in samples that were heterozygous at the expressed SNP and the risk variant. For each plate, the average allele ratio from all genomic DNA samples was used as a correction factor for all genomic DNA and cDNA allele ratios, since this can be assumed to reflect a perfect 1:1 ratio of the two alleles and can therefore be used to correct for any inequalities in allelic representation specific to the assay [Bray et al., 2003b]. The average of the four corrected allele ratios for genomic DNA and cDNA from each sample was calculated and used for statistical comparisons. Any samples showing poor reproducibility in cDNA allele ratios (standard deviation/mean > 0.25) were excluded from further analyses.

### Assessment of Association Between Schizophrenia Risk Alleles and Allele‐Specific Expression

Predicted haplotypes between the schizophrenia risk variants (rs11191419 and ch10_104957618_I) and the expressed SNP for each gene were calculated based on combined genotype data from the 116 adult and 95 fetal samples using Haploview 4.2 software [Barrett et al., 2005]. Predicted haplotype frequencies were used to infer phase between the risk alleles of rs11191419/ch10_104957618_I and the alleles of the exonic SNPs, so that the effect of the risk alleles on gene expression (i.e., up‐ or downregulation) could be determined, as described previously [Bray et al., 2005]. Specifically, for samples that were heterozygous at both the exonic SNP and a risk variant, we calculated the frequency of the two possible diplotypes constructed from the two alleles of the exonic SNP and the two alleles of the risk variant on the basis of predicted haplotype frequencies and the assumption of Hardy Weinberg equilibrium using the equations:

Frequency diplotype 1=2×frequencyhaplotypeA×frequencyhaplotypeB

Frequency diplotype 2=2×frequencyhaplotypeC×frequencyhaplotypeD

The probability that an individual who is heterozygous at both the exonic SNP and the risk variant is carrying diplotype 1 (comprising haplotypes A and B, rather than haplotypes C and D) is, therefore, calculated by dividing the predicted frequency of diplotype 1 by the combined frequency of both possible diplotypes.

Due to strong linkage disequilibrium in the region, the risk alleles of rs11191419 and ch10_104957618_I would nearly always be carried on the same chromosome as one of the alleles of each exonic SNP. As an initial test of whether the risk alleles were associated with a relative increase or decrease in the allelic expression of each gene, we therefore compared cDNA allele ratios in samples that were heterozygous for each risk variant (where the risk alleles would usually be carried on the same chromosome as one of the expressed alleles and the non‐risk alleles would be usually carried on the same chromosome as the alternative expressed allele) with the allele ratios observed in genomic DNA (representing a true 1:1 ratio of the two alleles). As a more specific test of whether risk genotype could account for altered *cis*‐regulation of each candidate gene, we also compared cDNA allele ratios between risk allele heterozygotes (where any *cis*‐regulatory effects of that variant will differ) and homozygotes (where any *cis*‐regulatory effects of the variant will be the same), as performed previously [Bray et al., 2003a, 2005; Hill and Bray, 2012]. Due to small numbers of homozygotes for rs11191419 in assayed exonic heterozygotes, these latter analyses were restricted to comparisons between ch10_104957618_I genotypes. All comparisons were performed by *t*‐tests using SPSS 22.0 software. Where differences in variance were detected between comparison groups (Levene's test *P* < 0.05), we used *t*‐tests that assumed unequal variance. All tests were two‐tailed and *P‐*values < 0.05 were considered to be significant. As a more stringent measure of the significance of each finding, we additionally applied a Bonferroni correction for the number of tests performed in each analysis. For the comparisions between allele ratios in cDNA from risk allele heterozygotes and those in genomic DNA, we corrected observed *P*‐values for 32 tests (assessing the effects of heterozygosity at two risk variants on the allelic expression of four genes in four brain tissues). For the comparisons between cDNA ratios observed in heterozygotes and homozygotes for ch10_104957618_I, we corrected observed *P*‐values for 16 tests (assessing the effect of ch10_104957618_I genotype on four genes in four brain tissues).

## RESULTS

One hundred and sixteen adult and 95 fetal human brains were initially genotyped for schizophrenia risk SNP rs11191419 and indel ch10_104957618_I (rs202213518), as well as exonic SNPs in *BORCS7* (rs4917985), *AS3MT* (rs1046778), *CNNM2* (rs2275271), and *NT5C2* (rs3740387), which could serve as allele‐specific tags for the four candidate genes in heterozygous samples. Frequencies of the schizophrenia risk alleles in our samples approximated those observed in the control sample of the recent Schizophrenia Working Group of the Psychiatric Genomics Consortium [2014] GWAS. The risk (T) allele of rs11191419 had a reported frequency of 0.64 in the GWAS control samples and a frequency of 0.64 in our combined fetal and adult brain samples, while the risk (deletion) allele of ch10_104957618_I had a reported frequency of 0.92 in the GWAS control samples and a frequency of 0.90 in our samples.

Predicted haplotype frequencies in our entire brain collection allowed us to infer phase between the risk alleles of rs11191419/ch10_104957618_I and the alleles of the exonic SNPs used to assess the allelic expression of each candidate gene (see Materials and Methods). The strong linkage disequilibrium (D′) in the region meant that, in individuals who were heterozygous at a risk variant as well as an exonic variant, the risk allele would nearly always be carried on the same chromosome as a particular allele of the exonic SNP. An assessment of whether the risk alleles were associated with a general increase or decrease in the allelic expression of each candidate gene could, therefore, be made by comparing cDNA allele ratios in individuals who were heterozygous for the risk variant with allele ratios observed in genomic DNA (representing the true 1:1 ratio of the two alleles) [e.g., Bray et al., 2003a, 2005].

**Figure 2:**
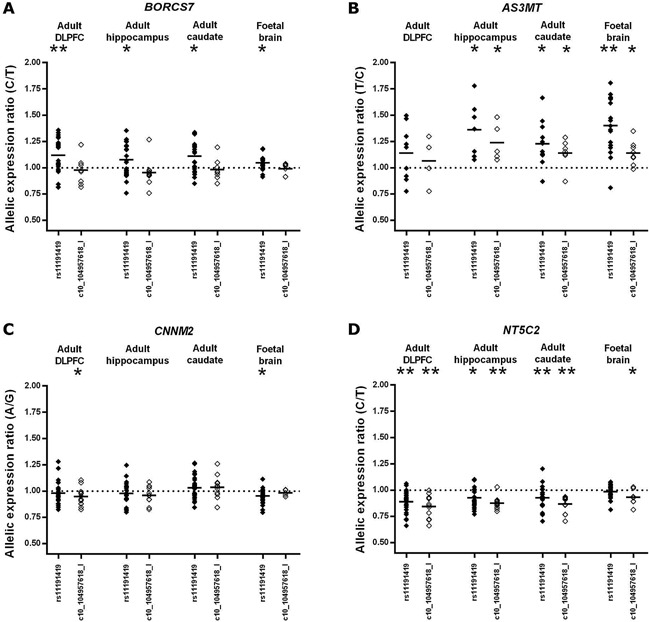
Allelic expression of *BORCS7* (A), *AS3MT* (B), *CNNM2* (C), and *NT5C2* (D) in heterozygotes for schizophrenia risk variants rs11191419 and ch10_104957618_I. Allelic expression ratios are calculated by dividing measures of the expressed allele that is generally in phase with the schizophrenia risk alleles by measures of the expressed allele that is generally in phase with the non‐risk alleles for each susceptibility variant. All raw cDNA ratios are divided by the average allele ratio in genomic DNA (representing the true 1:1 allele ratio) to correct for any inequalities in allelic representation specific to each assay. Data points represent the average of four corrected measures of cDNA allele ratio per sample. Mean corrected cDNA allele ratios are indicated by horizontal lines. The dotted horizontal line indicates the mean genomic DNA (1:1) ratio of the two alleles. Comparisons between cDNA allele ratios in heterozygotes for the risk variants and allele ratios in genomic DNA: *< 0.05, **P < 0.05 when Bonferroni corrected for 32 tests.

Allelic expression data at SNP rs4917985, used to tag *BORCS7*, in heterozygotes for rs11191419 and ch10_104957618_I, are shown in Figure 2A. The C‐allele of rs4917985 was predicted to be in phase with the risk (T‐) allele of rs11191419 on >99% of occasions when the subject was heterozygous at both loci. Allele ratios in cDNA from rs11191419 heterozygotes indicated a mean increase in expression of the allele carried on the same chromosome as the risk allele, relative to that carried with the non‐risk allele, in all assayed tissues (DLPFC: 12%, hippocampus: 8%, caudate: 11%, fetal brain 5%). cDNA allele ratios in all assayed tissues differed significantly from allele ratios observed in genomic DNA (all *P* < 0.05). Although *P*‐values survived Bonferroni correction for 32 independent tests only in the DLPFC (*P* = 0.001, corrected *P* = 0.032), *BORCS7* cDNA ratios did not differ significantly from those obtained from rs11191419 heterozygotes in any other adult brain region (all *P *> 0.05). The C‐allele of rs4917985 was also predicted to be in phase with the risk (deletion) allele of ch10_104957618_I on >99% of occasions when the subject was heterozygous at both loci. However, allele ratios in cDNA from ch10_104957618_I heterozygotes were close to the 1:1 ratio of equal allelic expression, and did not significantly differ from those observed in genomic DNA in any tissue.

Allelic expression data at SNP rs1046778, used to tag *AS3MT*, in heterozygotes for rs11191419 and ch10_104957618_I, are shown in Figure 2B. The T‐allele of rs1046778 was predicted to be in phase with the risk (T‐) allele of rs11191419 on >94% of occasions when the subject was heterozygous at both loci. As for *BORCS7*, cDNA allele ratios in rs11191419 heterozygotes indicated a mean increase in expression of the *AS3MT* allele carried on the same chromosome as the risk allele, compared to that carried with the non‐risk allele, in all assayed tissues (DLPFC: 14%, hippocampus: 36%, caudate: 23%, fetal brain 40%). cDNA allele ratios differed significantly from allele ratios observed in genomic DNA in fetal brain and in adult hippocampus and caudate (*P* < 0.05 in all tissues). Although *P*‐values survived Bonferroni correction for 32 independent tests only in fetal brain (*P* = 1.12 × 10^−5^, corrected *P* = 0.00036), *AS3MT* cDNA ratios did not differ significantly from those observed in rs11191419 heterozygotes in adult hippocampus or caudate (*P *> 0.05). The T‐allele of rs1046778 was predicted to be in phase with the risk (deletion) allele of ch10_104957618_I on >99% of occasions when the subject was heterozygous at both loci. Relative overexpression of the *AS3MT* allele in phase with the risk allele was less pronounced than in rs11191419 heterozygotes (DLPFC: 7%, hippocampus: 24%, caudate: 14%, fetal brain 14%). While allele ratios in ch10_104957618_I heterozygotes differed significantly between cDNA and genomic DNA in hippocampus, caudate and fetal brain (all *P* < 0.05), no observation survived Bonferroni correction.

Allelic expression data at SNP rs2275271, used to tag *CNNM2*, in heterozygotes for rs11191419 and ch10_104957618_I, are shown in Figure 2C. The A‐allele of rs2275271 was predicted to be in phase with the risk (T‐) allele of rs11191419 on >98% of occasions when the subject was heterozygous at both loci. However, unlike cDNA allele ratios in rs11191419 heterozygotes for *BORCS7* and *AS3MT*, those for *CNNM2* were close to the 1:1 ratio of equal allelic expression, only differing significantly from those in genomic DNA in fetal brain (*P* = 0.012), where a small (5%) relative decrease in the mean expression of the allele generally in phase with the risk allele of rs11191419 was observed. The A‐allele of rs2275271 was predicted to be in phase with the risk (deletion) allele of ch10_104957618_I on >99% of occasions when the subject was heterozygous at both loci. Heterozygotes for ch10_104957618_I also displayed little allelic expression imbalance of *CNNM2*, with cDNA allele ratios only differing significantly from those in genomic DNA in the adult DLPFC, where a mean 5% decrease in expression of the allele usually in phase with the risk allele was observed (*P* = 0.015). No allelic expression imbalance of *CNNM2* associated with heterozygosity for the assayed schizophrenia risk variants survived Bonferroni correction.

Allelic expression data at SNP rs3740387, used to tag *NT5C2*, in heterozygotes for rs11191419 and ch10_104957618_I, are shown in Figure 2D. The C‐allele of rs3740387 was predicted to be in phase with the risk (T‐) allele of rs11191419 on >98% of occasions when the subject was heterozygous at both loci. In rs11191419 heterozygotes, expression of the *NT5C2* allele generally in phase with the risk allele was reduced in all assayed adult brain regions (mean DLPFC: 11%, hippocampus: 7%, caudate: 7%), with cDNA allele ratios differing significantly from those in genomic DNA (all *P* < 0.05). Observed *P*‐values survived Bonferroni correction for 32 independent tests in the adult DLPFC (*P* = 6.29 × 10^−7^, corrected *P* = 2.01 × 10^−5^) and caudate (*P* = 0.001, corrected *P* = 0.032), with a less significant imbalance of allelic expression observed in the hippocampus (*P* = 0.003, corrected *P* = 0.083). The C‐allele of rs3740387 was predicted to be in phase with the risk (deletion) allele of ch10_104957618_I on >99% of occasions when the subject was heterozygous at both loci. Expression of the *NT5C2* allele in phase with the ch10_104957618_I risk allele was also reduced in all assayed adult brain regions (mean DLPFC: 15%, hippocampus: 12%, caudate: 13%), with highly significant differences in allele ratios observed between cDNA and genomic DNA that survived Bonferroni correction in all areas (DLPFC: *P* = 1.03 × 10^−4^, corrected *P* = 0.003; hippocampus: *P* = 2.64 × 10^−6^, corrected *P* = 8.46 × 10^−5^; caudate *P* = 8.11 × 10^−5^, corrected *P* = 0.0025). Unlike cDNA ratios in rs11191419 heterozygotes, those in ch10_104957618_I heterozygotes also significantly differed from genomic DNA in fetal brain (*P* = 0.05), with the risk allele again associated with reduced *NT5C2* allelic expression, although this latter observation did not survive Bonferroni correction.

**Figure 3:**
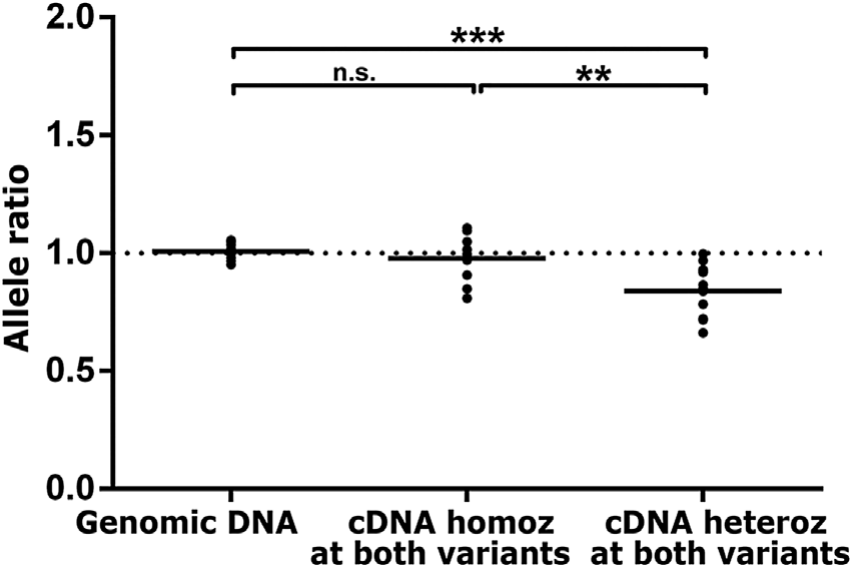
Allele ratios at expressed *NT5C2* SNP rs3740387 in the DLPFC of adult subjects who are homozygous at both rs11191419 and ch10_104957618_I (6 M, 3 F, average age = 64 years) and in adult subjects who are heterozygous at both variants (9 M, 4 F, average age = 70 years). Data points represent the average of four corrected measures of allele ratio in genomic DNA or cDNA per sample. Mean corrected allele ratios are indicated by horizontal lines. The dotted horizontal line indicates the mean genomic DNA (1:1) ratio of the two alleles. Allele ratios in cDNA from subjects who are homozygous at both risk variants do not significantly differ from those in genomic DNA. Allele ratios in cDNA from subjects who are heterozygous at both risk variants differ significantly from those in genomic DNA and from those in cDNA from homozygotes at both variants. **< 0.01, ***P < 0.001.

Although comparisons between cDNA and gDNA allele ratios in heterozygous risk allele carriers under conditions of high‐linkage disequilibrium allow an assessment of whether the risk allele is associated with a general increase or decrease in allelic expression [Bray et al., 2003a, 2005; Hill and Bray, 2012], they do not specifically test whether genotype at the risk variant could directly account for altered *cis*‐regulation of the gene. For this, it is necessary to compare cDNA allele ratios in heterozygotes for the risk variant (where any *cis*‐regulatory effects of the two alleles will differ) with those in homozygotes for the risk variant (where any *cis*‐regulatory effects of the variant will be equal) [Bray et al., 2003a, 2005; Williams et al., 2011; Hill and Bray, 2012]. Small genotype groups precluded such an assessment of *cis*‐regulatory effects of rs11191419, but genotype at ch10_104957618_I was associated with significant effects on the allelic expression of *BORCS7* in fetal brain, adult DLPFC, adult hippocampus and adult caudate, *AS3MT* in the fetal brain and adult caudate, and *NT5C2* in the fetal brain, adult DLPFC and adult hippocampus. *P*‐values survived Bonferroni correction for 16 tests for *BORCS7* in the adult hippocampus (*P* = 0.001, corrected *P* = 0.016) and fetal brain (*P* = 0.002, corrected *P* = 0.032), *AS3MT* in the fetal brain (*P* = 0.002, corrected *P* = 0.032), and *NT5C2* in the adult DLPFC (*P* = 0.003, corrected *P* = 0.048). Mean cDNA allele ratios at the four candidate genes in ch10_104957618_I heterozygotes and homozygotes are shown in Table I. These analyses showed that the risk allele of ch10_104957618_I is associated with a downregulation of both *BORCS7* and *AS3MT*, reducing the general overexpression of these genes associated with the risk allele of rs11191419, with less allelic expression imbalance observed in heterozygotes for ch10_104957618_I than in homozygotes. This is consistent with the initial comparisons between allele ratios in cDNA and genomic DNA, which showed less pronounced allelic expression imbalance of *BORCS7* and *AS3MT* in ch10_104957618_I heterozygotes than in rs11191419 heterozygotes. In contrast, the risk alleles of rs11191419 and ch10_104957618_I appear both to be associated with reduced expression of *NT5C2*. Indeed, genotype at rs11191419 and ch10_104957618_I could largely account for observed allelic expression imbalance of *NT5C2* in the adult DLPFC (Fig. 3), where samples that were heterozygous at both risk loci showed a mean 16% reduction in *NT5C2* allelic expression, while homozygotes at both risk loci displayed cDNA allele ratios close to the genomic 1:1 ratio (comparison between cDNA ratios in heterozygotes and homozygotes at both loci: *P* = 0.007).

**Table I:** Average Corrected cDNA Allele Ratios at Expressed SNPs in *BORCS7*, *AS3MT*, *CNNM2*, and *NT5C2* According to Genotype at Schizophrenia Risk Variant ch10_104957618_I

Gene (expressed SNP)	Genotype at ch10_104957618_I	Adult DLPFC	Adult hippocampus	Adult caudate	Fetal whole brain
*BORCS7* (rs4917985; C/T^a^)	Heterozygous	0.99	0.95	0.98	0.99
	Homozygous	1.17	1.12	1.15	1.06
	*P* het versus hom	**0.006**	**0.001** ^*^	**0.004**	**0.002** ^*^
					
*AS3MT* (rs1046778; T/C)	Heterozygous	1.07	1.24	1.14	1.14
	Homozygous	1.25	1.52	1.31	1.42
	*P* het versus hom	0.235	0.187	**0.034**	**0.002** ^*^
					
*CNNM2* (rs2275271; A/G)	Heterozygous	0.95	0.96	1.04	0.98
	Homozygous	0.99	0.98	1.02	0.95
	*P* het versus hom	0.095	0.629	0.527	0.342
					
*NT5C2* (rs3740387; C/T)	Heterozygous	0.85	0.88	0.87	0.93
	Homozygous	0.94	0.95	0.93	0.99
	*P* het versus hom	**0.003** ^*^	**0.014**	0.072	**0.016**

^a^ Allele ratios at each expressed SNP were calculated by dividing measures of the allele generally in phase with the schizophrenia risk alleles by measures of the allele generally in phase with the non‐risk alleles, as indicated.

^*^ *P*‐values surviving Bonferroni correction for 16 tests.

Uncorrected *P*‐values < 0.05 are indicated in bold.

## DISCUSSION

Variants on chromosome 10q24.32‐q24.33 exhibit robust association with schizophrenia [Schizophrenia Psychiatric Genome‐Wide Association Study Consortium, 2011; Aberg et al., 2013; Ripke et al., 2013; Schizophrenia Working Group of the Psychiatric Genomics Consortium, 2014], but, like many regions implicated by GWAS, the actual susceptibility genes cannot be easily resolved through genetic data alone. Using a highly sensitive method for assessing variable *cis*‐effects on gene expression [Yan et al., 2002; Bray et al., 2003a,2003b], we have found that several of the principal candidate genes at this locus exhibit altered *cis*‐regulation in the developing and adult human brain in association with the most strongly supported schizophrenia risk variants. Largest and most consistent effects were observed on *BORCS7*, *AS3MT*, and *NT5C2*, providing functional support for these as genuine susceptibility genes for schizophrenia.

Our data indicate a complex pattern of *cis*‐regulation at the chromosome 10q24 locus. The indel ch10_104957618_I (rs202213518) is located 4,555 bp upstream of the predicted transcription start site of *NT5C2* transcript variant 1 (NM_012229). ENCODE ChIP‐seq data indicate that ch10_104957618_I resides in an H3K27ac‐marked region that is bound by multiple transcription factors, suggesting direct effects of this variant on *NT5C2* transcription. However, genotype at this variant was also found to influence the allelic expression of *BORCS7* and *AS3MT*, with the risk allele (in contrast to that of rs11191419) associated with reduced allelic expression of these genes. Similarly, heterozygosity for rs11191419, located within 2 kb of the transcriptional start site of *BORCS7*, was found to be associated with allelic expression imbalance of *BORCS7*, *AS3MT*, and *NT5C2*. This could result from long‐range enhancer effects of these variants (or variants in linkage disequilibrium with them), transcriptional interference on adjacent gene expression, or linkage disequilibrium with other functional variants at the chromosome 10q24 locus.

It appears that the risk alleles of rs11191419 and ch10_104957618_I have opposing effects on the expression of both *BORCS7* and *AS3MT*, with the risk (T‐) allele of rs11191419 associated with increased allelic expression and the risk (deletion) allele of ch10_104957618_I associated with decreased allelic expression of these genes. This is consistent with the risk allele of ch10_104957618_I conferring susceptibility to schizophrenia through effects on a different gene, such as *NT5C2*. However, the risk allele of ch10_104957618_I appears insufficient to fully counteract the increased expression of *AS3MT* associated with the risk allele of rs11191419, with allele ratios in cDNA from ch10_104957618_I heterozygotes remaining significantly higher than the genomic 1:1 ratio in most assayed tissues. In contrast, both risk alleles of rs11191419 and ch10_104957618_I are associated with reduced allelic expression of *NT5C2*, and appear to account for the majority of *cis*‐regulatory effects on this gene observed in the adult DLPFC. It is possible that the strong association between rs11191419 and schizophrenia is due to it indexing functional risk variation affecting the regulation of multiple genes at the locus.

Although only some observations survived Bonferroni correction for multiple testing, we urge caution in drawing conclusions as to the relative importance of each finding on the basis of *P*‐values alone, since the number of subjects differed between analyses due to differences in expressed allele frequency between candidate genes and the availability of brain tissue from each region. As can be seen in Figure 2, for *BORCS7*, *AS3MT*, and *NT5C2* at least, we observed a general consistency in the effects of risk variant heterozygosity on allelic expression across the brain tissues analyzed.

There are no previous data assessing the impact of ch10_104957618_I genotype on gene expression. However, our findings for rs11191419 appear consistent with existing data generated by eQTL and bioinformatic approaches. The Schizophrenia Working Group of the Psychiatric Genomics Consortium study [2014] included analyses that sought to relate credible GWAS risk variants to genome‐wide eQTL data, finding rs11191419 to be in strong linkage disequilibrium (r^2^ = 0.85) with an eQTL SNP (rs7096169) influencing *AS3MT* expression in blood. Using several brain eQTL datasets, Roussos et al. [2014] identified SNPs influencing *BORCS7 (C10ORF32)*, *AS3MT*, *WBP1L*, and *NT5C2* expression that are in linkage disequilibrium with rs7085104, identified in an earlier GWAS of schizophrenia [Ripke et al., 2013], which we found to be in strong linkage disequilibrium (r^2 ^= 0.79) with rs11191419 in the samples genotyped in the present study. These authors also assessed whether schizophrenia‐associated eQTLs were located in predicted *cis*‐regulatory elements (CREs); expression of *BORCS7* and *AS3MT* was reported to be influenced by SNPs within individual CRE, while expression of *NT5C2* was associated with SNPs in 14 such elements [Roussos et al., 2014]. Most recently, a genome‐wide analysis of DNA methylation QTL in the human fetal brain has indicated that rs7085104 and SNPs in linkage disequilibrium with it are QTL for methylation probes within *AS3MT* [Hannon et al., 2016], consistent with our observation of a large allelic expression imbalance of *AS3MT* in association with rs11191419 heterozygosity in the fetal brain.

Our study is the first to specifically explore effects of chromosome 10q24 schizophrenia risk variants on gene expression in the human fetal brain. Microarray data indicate that *AS3MT* and *NT5C2* are both expressed at a higher level in the prenatal human brain compared to that of the adult [Kang et al., 2011; Birnbaum et al., 2015]. We find that heterozygosity for rs11191419 is associated with particularly pronounced allelic expression imbalance of *AS3MT* in the fetal brain, with an average 40% increase in expression of the *AS3MT* allele that is generally carried on the same chromosome as the risk allele. We find no evidence that *NT5C2* expression is influenced by rs11191419 genotype in fetal brain, but we do observe small effects of ch10_104957618_I genotype on *NT5C2* allelic expression at this early stage of development. These findings would therefore appear consistent with an early neurodevelopmental component to schizophrenia [Murray and Lewis, 1987; Weinberger, 1987], although the observed persistence of effects in the adult brain suggests an ongoing risk mechanism.

A limitation of our study is that it focused on a restricted number of positional candidate genes at the chromosome 10q24.32‐q24.33 locus. Although we selected the four candidates flanked by the two best supported risk variants (Fig. 1), extended linkage disequilibrium and the possibility of long‐range effects on gene regulation [Sanyal et al., 2012] implicate several other known genes in the region (e.g., *WBP1L*, *CYP17A1*, *INA*, *PCGF6*). In addition, through our use of exonic SNPs that typically tag multiple alternative transcripts of a given gene, we might underestimate *cis*‐regulatory effects on individual transcripts, while missing effects on any transcripts that do not include those SNPs. Some of these limitations could be overcome by RNA sequencing, which can be used to measure allele‐specific (as well as total) expression of individual transcripts on a genome‐wide scale.

The neural functions of the genes implicated in this study remain to be fully elucidated. *BORCS7* encodes BLOC‐1‐related complex subunit 7 (Diaskedin), part of the recently described BLOC‐1‐related complex, which has been implicated in lysosomal function and cell migration [Pu et al., 2015]. *AS3MT* encodes arsenic methyltransferase, which has a known role in arsenic metabolism [Sumi and Himeno, 2012], although its functions in the brain are currently unclear. *NT5C2* encodes a cytosolic purine 5′‐nucleotidase (cytosolic 5′‐nucleotidase II, or cN‐II) involved in cellular purine metabolism [Itoh, 2013]. A purinergic hypothesis of schizophrenia has been proposed to explain neurochemical as well as neurodevelopmental aspects of the disorder [Lara and Souza, 2000].

In summary, we have provided an assessment of *cis*‐regulatory effects associated with schizophrenia risk variants in a region of extensive linkage disequilibrium on chromosome 10q24. We report altered *cis*‐regulation of *BORCS7*, *AS3MT*, and *NT5C2* in association with schizophrenia risk variation, implicating these as genuine schizophrenia susceptibility genes at the locus. Further characterization of these genes in the developing and adult brain is now warranted in order to understand how perturbations in their expression might confer risk for schizophrenia.

## Supporting information

Additional supporting information may be found in the online version of this article at the publisher's web‐site.

**Table S1**. Demographics for subjects heterozygous for schizophrenia risk variants assayed for each candidate gene. * Values represent number of males (M), females (F), average age (range). Age is in years for adult samples, and in post‐conception days for fetal samples.

**Table S2**. Primers used to amplify expressed SNPs and schizophrenia risk variants at the chromosome 10q24 locus.
