# Supplementary material for: Genome‐wide significant schizophrenia risk variation on chromosome 10q24 is associated with altered cis‐regulation of BORCS7, AS3MT, and NT5C2 in the human brain
Source: Am J Med Genet B Neuropsychiatr Genet. 2016 Mar 22;171(6):806–14. doi: 10.1002/ajmg.b.32445 (PMC4988385; doi:10.1002/ajmg.b.32445)
Supplement: Supplementary file 1 — Table S1. Demographics for subjects heterozygous for schizophrenia risk variants assayed for each candidate gene. * Values represent number of males (M), females (F), average age (range). Age is in years for adult samples, and in post‐conception days for fetal samples. [file AJMG-171-806-s001.docx]

|  | | | | | | | |  |
| --- | --- | --- | --- | --- | --- | --- | --- | --- |
|  | **Adult DLPFC** | | **Adult Hippocampus** | | **Adult Caudate** | | **Fetal brain** | |
|  | **hets rs11191419** | **hets chr10_104957618_I** | **hets rs11191419** | **hets chr10_104957618_I** | **hets rs11191419** | **hets chr10_104957618_I** | **hets rs11191419** | **hets chr10_104957618_I** |
| ***BORCS7*** | 19M, 9F; 71.6 (18–92) | 8M, 1F; 65.9 (18–88) | 17M, 7F; 76.5 (54–96) | 7M, 4F; 76.4 (54–96) | 12M, 10F; 77.9 (42–96) | 4M, 4F; 81 (66–96) | 13M, 11F; 106.3 (91–153) | 4M, 4F; 104.3 (91–133) |
| ***AS3MT*** | 6M, 2F; 70.9 (54–90) | 8M, 1F; 67 (54–78) | 5M, 2F; 79.9 (67–92) | 3M, 2F; 78 (67–92) | 4M, 7F; 78.8 (55–92) | 3M, 3F; 82 (69–92) | 7M, 10F; 105 (91–153) | 5M, 3F; 107.4 (91–133) |
| ***CNNM2*** | 21M, 12F; 72.8 (18–96) | 11M, 5F; 70.1 (18–96) | 16M, 9F; 77.8 (54–96) | 9M, 5F; 75.6 (54–96) | 18M, 12F; 75.6 (18–96) | 7M, 5F; 74.1 (18–96) | 16M, 13F; 113 (91–161) | 3M, 3F; 119.2 (93–154) |
| ***NT5C2*** | 20M, 11F; 74.5 (18–96) | 10M, 4F; 71.3 (18–96) | 13M, 10F; 78.9 (54–96) | 7M, 6F; 77.5 (54–96) | 14M, 11F; 74.9 (18–96) | 7M, 5F; 74.1 (18–96) | 10M, 10F; 107.7 (91–153) | 4M, 3F; 111.4 (93–133) |
|  |  |  |  |  |  |  |  |  |
|  | | | | | | | | |

**Supplementary Table S1**. Demographics for subjects heterozygous for schizophrenia risk variants assayed for each candidate gene. * Values represent number of males (M), females (F), average age (range). Age is in years for adult samples, and in post-conception days for fetal samples.
