# Supplementary material for: Genome‐wide significant schizophrenia risk variation on chromosome 10q24 is associated with altered cis‐regulation of BORCS7, AS3MT, and NT5C2 in the human brain
Source: Am J Med Genet B Neuropsychiatr Genet. 2016 Mar 22;171(6):806–14. doi: 10.1002/ajmg.b.32445 (PMC4988385; doi:10.1002/ajmg.b.32445)
Supplement: Supplementary file 2 — Table S2. Primers used to amplify expressed SNPs and schizophrenia risk variants at the chromosome 10q24 locus. [file AJMG-171-806-s002.docx]

| **Variant** | **Gene** | **MAF** | **Reason for amplifying** | **Forward primer (5’-3’)** | **Reverse primer (5’-3’)** | **Extension primer (5’-3’)** | **Amplicon size (bp)** |
| --- | --- | --- | --- | --- | --- | --- | --- |
|  |  |  |  |  |  |  |  |
| rs4917985 | *BORCS7* | 0.37 | Allelic expression assay | TGTTGTAGTTCAGGTCTTCATTGA | TGCCTTTTCAGATCCTTCACA | TGCCTTTTCAGATCCTTCACATC | 228 |
| rs1046778 | *AS3MT* | 0.31 | Allelic expression assay | CAAGAAGCAGGAAAGGCATC | TTGGCAATCTTTTTGCATGA | GATCTTTTGCATAGCACCTT | 165 |
| rs2275271 | *CNNM2* | 0.39 | Allelic expression assay | CGGGAACGAAAGCAAGATT | GTTGCTAGGAAACGGTGCAT | TGTGGTGATATTTTAACCTTCATCTC | 104 |
| rs3740387 | *NT5C2* | 0.40 | Allelic expression assay | CTCCCAACCTCTTCCCACTG | GGACCTCGTTTGTTCCTGTG | CAGGAAATTACACACTGCCATGA | 159 |
| rs11191419 | - | 0.36 | Risk SNP genotyping | TGAATCTAGACACTTGGAAGAGG | GAAGTTGAGGCGGGAGGAT | CCACGCCCGGCTAATTTTTG | 89 |
| ch10_104957618_I | *-* | 0.1 | Risk indel Sanger sequencing | GAGTTAGGATCGGGTGAGGG | GTCCGGTCATAGCTCACTGA | - | 346 |

**Supplementary Table S2**. Primers used to amplify expressed SNPs and schizophrenia risk variants at the chromosome 10q24 locus
